# Unilateral Vocal Cord Paralysis Diagnosed with Dynamic Digital Radiography

**DOI:** 10.3390/diagnostics15192502

**Published:** 2025-10-01

**Authors:** Michaela Cellina

**Affiliations:** Radiology Department, ASST FBF SACCO, Fatebenefratelli Hospital, Piazza Principessa Clotilde 3, 20121 Milan, Italy; michaela.cellina@asst-fbf-sacco.it

**Keywords:** vocal cord paralysis, DDR, dynamic digital radiography, laryngeal imaging, flat-panel detector

## Abstract

Flexible laryngoscopy (FL) is the standard diagnostic tool for vocal cord paralysis (VCP), but it involves patient discomfort, and its interpretation is subjective and operator-dependent. Dynamic digital radiography (DDR) is a novel imaging technique that acquires high-resolution sequential radiographs at a low radiation dose. While DDR has been widely applied in chest and diaphragmatic imaging, its use for laryngeal motion analysis has been poorly investigated. We present the case of a 50-year-old male referred for Computed Tomography (CT) of the neck and chest for suspected vocal cord paralysis. The referring physician did not specify the side of the suspected paralysis. Due to a language barrier and the absence of prior documentation, a detailed history could not be obtained. To assess vocal cord motion, we performed, for the first time in our Institution, a DDR study of the neck. During phonation maneuvers, DDR demonstrated fixation of the left vocal cord in an adducted paramedian position. CT confirmed this finding and did not highlight any further anomaly. This case demonstrates the feasibility of DDR as a low-cost, low-dose, non-invasive technique for functional evaluation of the larynx and may represent a valuable complementary imaging tool in laryngeal functional assessment.

**Figure 1:**
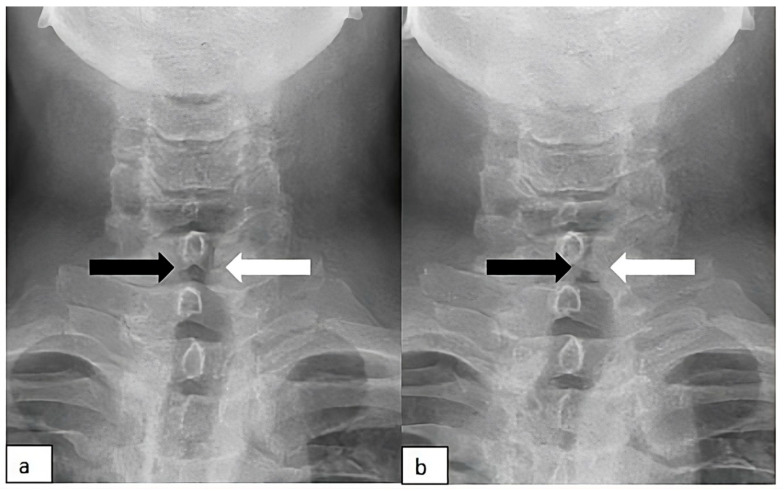
To assess vocal cord function, a DDR acquisition was acquired (AeroDR TX, Konica Minolta Inc., Tokyo, Japan; Konica Minolta, Tokyo, Japan). The patient was positioned in an anterior–posterior sitting position, with the acanthiomeatal line used as reference, 10° head elevation, and a 5° caudal tube angle to optimize visualization of the laryngeal structures, as previously described [1,2], with the following acquisition parameters: 90 kV, 200 mA, pulse time of 2.5 ms, and total imaging time of 10 s, corresponding to an effective dose of 1.15 mGy. The phonation maneuver involves the utterance of “/i/”. The whole acquisition is in the Supplementary Materials. Images obtained from the sequential dynamic acquisition obtained with DDR: (**a**) Neutral condition; (**b**) phonation. While the right vocal cord (black arrow) changes its position during the phonation with normal abduction and adduction, the left vocal cord (white arrow) is fixed and constantly in an adducted paramedian position. The left cord remained immobile throughout all phases of respiration and phonation, consistent with unilateral left vocal cord paralysis. Flexible laryngoscopy (FL) is the standard diagnostic tool for vocal cord paralysis (VCP), but it involves patient discomfort, and its interpretation is subjective and operator-dependent. Additionally, the FL examination may be restricted for patients with unfavorable laryngeal anatomy, such as a strong gag reflex, epiglottis deformation, or pseudo vocal cord vocalization, in which the arytenoid cartilage covering the vocal cords during vocalization, thus making the visualization of vocal cord movements difficult [3]. Dynamic digital radiography (DDR) is a novel imaging modality that acquires sequential X-ray images at 15 frames/sec using a flat-panel detector [4] and generates high-temporal-resolution dynamic images at a low radiation dose [5]. DDR captures the movement of anatomical structures in physiological and pathological conditions, providing functional information that cannot be obtained from static imaging [6]. While this technique has been widely applied in chest and diaphragmatic imaging [7,8,9,10], its use for laryngeal motion analysis has been poorly investigated [1,2]. DDR may serve as a valuable adjunct in the assessment of vocal cord motion, particularly in situations where FL is contraindicated or yields inconclusive findings. Potential indications include patients with anatomical limitations, intolerance to endoscopic examination, infectious concerns that preclude laryngoscopy, or communication barriers that limit conventional clinical evaluation. In addition, DDR can provide objective, image-based documentation of laryngeal motion for follow-up or multidisciplinary discussions. Although DDR involves ionizing radiation, the exposure is low and comparable to that of conventional chest radiography. Therefore, neck DDR can have a role in the diagnostic workflow of suspected VCP, particularly in cases where laryngoscopic evaluation is unavailable or inconclusive.

**Figure 2:**
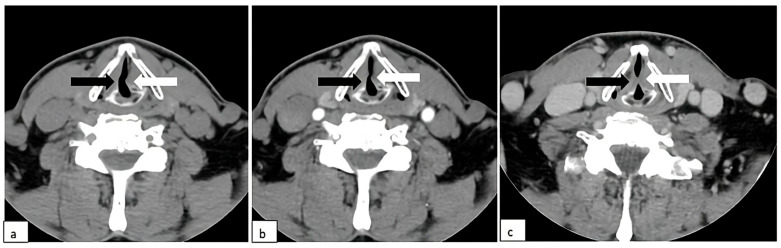
Neck CT: (**a**) unenhanced scan; (**b**) contrast-enhanced scan; (**c**) phonation. Paralyzed left vocal cord (white arrow), fixed and constantly in an adducted paramedian position. Normal appearance of the right vocal cord with adduction in phonation (black arrows) (**c**).

## Data Availability

Not applicable.

## Supplementary Materials

The following supporting information can be downloaded at: https://www.mdpi.com/article/10.3390/diagnostics15192502/s1 and https://eu-west-1.protection.sophos.com/?d=google.com&u=aHR0cHM6Ly9kcml2ZS5nb29nbGUuY29tL2ZpbGUvZC8xSHFWajZhdjhBQmVIREkwQU5oY0dsY3I2SDY4a19KU3kvdmlldz91c3A9ZHJpdmVzZGs=&i=Njg2NDAyZmRkOGNkNWY0NzMyNWM3OGRj&t=ai9FUVErK3FGVVE4ZE02dXJCQ0N3clpNak1vNUhaZ2dPY05ZL3N0dVVKVT0=&h=62e51b2c43ea40f7af38876be29c314e&s=AVNPUEhUT0NFTkNSWVBUSVbpOfxyJIr0dIT_jBA7YBe_H29WJBnFZkgn_q1-YGGpQg: The whole DDR acquisition of the neck performed in the anterior–posterior projection. The acquisition was performed in rest conditions and during “/i/” phonation to assess the vocal cord mobility. DDR demonstrated persistent fixation of the left vocal cord in an adducted paramedian position. This finding is consistent with unilateral vocal cord paralysis. The right vocal cord showed normal dynamic mobility.

## Abbreviations

The following abbreviations are used in this manuscript:

FL	Flexible Laryngoscopy
VCP	Vocal Cord Paralysis
DDR	Dynamic Digital Radiography
